# Computer-Aided Diagnoses for Sore Throat Based on Dynamic Uncertain Causality Graph

**DOI:** 10.3390/diagnostics13071219

**Published:** 2023-03-23

**Authors:** Xusong Bu, Mingxia Zhang, Zhan Zhang, Qin Zhang

**Affiliations:** 1 Department of Computer Science and Technology, Tsinghua University, Beijing 100084, China; 2 Otorhinolaryngology Head & Neck Surgery, Xuan Wu Hospital of the Capital Medical University, Beijing 100053, China; 3 Institute of Nuclear and New Energy Technology, Tsinghua University, Beijing 100084, China

**Keywords:** causality, probability graph, sore throat, computer-aided diagnoses

## Abstract

The causes of sore throat are complex. It can be caused by diseases of the pharynx, adjacent organs of the pharynx, or even systemic diseases. Therefore, a lack of medical knowledge and experience may cause misdiagnoses or missed diagnoses in sore throat diagnoses, especially for general practitioners in primary hospitals. This study aims to develop a computer-aided diagnostic system to assist clinicians in the differential diagnoses of sore throat. The computer-aided system is developed based on the Dynamic Uncertain Causality Graph (DUCG) theory. We cooperated with medical specialists to establish a sore throat DUCG model as the diagnostic knowledge base. The construction of the model integrates epidemiological data, knowledge, and clinical experience of medical specialists. The chain reasoning algorithm of the DUCG is used for the differential diagnoses of sore throat. The system can diagnose 27 sore throat-related diseases. The model builder initially tests it with 81 cases, and all cases are correctly diagnosed. Then the system is verified by the third-party hospital, and the diagnostic accuracy is 98%. Now, the system has been applied in hundreds of primary hospitals in Jiaozhou City, China, and the degree of recognition for doctors to the diagnostic results of the system is more than 99.9%. It is feasible to use DUCG for the differential diagnoses of sore throat, which can assist primary doctors in clinical diagnoses and the diagnostic results are acceptable to clinicians.

## 1. Introduction

Sore throat is a common clinical symptom. Pharyngeal infection, trauma, ulcer, foreign body, malignant tumor, styloid process syndrome, and some systemic diseases manifest as sore throats of varying degrees [1,2,3]. Because the causes of sore throat are complex, it is necessary to make differential diagnoses. Lack of clinical experience may lead to missed diagnoses or misdiagnoses of sore throat, especially for doctors in primary hospitals. Therefore, using computer-aided technology to complete the diagnosis of sore throat is one of the solutions. Computer-aided diagnoses and decision-making systems can help doctors shorten diagnostic time, reduce missed diagnoses and misdiagnoses, and make the diagnoses as soon as possible [4,5,6,7]. Since 1970, various algorithms have been applied to computer-aided clinical diagnoses, including rule-based algorithms [8,9,10,11], case-based reasoning methods [12], machine learning methods [13,14,15,16,17,18,19], and probabilistic models [20,21,22,23]. However, those algorithms have some defects when used in computer-aided clinical diagnoses. Clinical diagnoses require a lot of knowledge, but the rule-based and case-based methods are challenging to manage a large amount of knowledge, existing problems of knowledge conflict, and reasoning inefficiency. The computer-aided clinical diagnostic system needs to be interpretable. Most machine learning algorithms have no explainable or weak interpretability. For example, the SVM and the neural network models are incomprehensible to doctors, and their algorithms cannot interpret how the diagnostic results are obtained. The Bayesian network is one of the probabilistic graphical models with the ability of interpretability. The training of conditional probability tables (CPTs) requires a lot of high-quality data sets. However, in reality, it is difficult for us to obtain a large number of high-quality medical records to train Bayesian networks, which affects the practical application of the Bayesian network in computer-aided clinical diagnoses. These shortcomings weaken doctors’ confidence in the diagnostic results. Therefore, those models are difficult to generalize in practical clinical applications.

The algorithms applied in computer-aided clinical decision-making need to have high diagnostic accuracy. Meanwhile, it also requires the capability of interpreting the results, and the diagnostic methods are in line with the diagnostic idea of clinical doctors.

Currently, the clinical diagnostic system can be divided into expert knowledge-based and neural network-based. The rule-based expert systems have explanatory ability, but their reasoning efficiency is not high, and they have difficulties in knowledge management. The neural network-based diagnostic systems have no interpretability to the diagnostic results. In this study, we developed an intelligent clinical diagnostic system based on the DUCG for sore throat diseases. It has high reasoning efficiency and the ability to explain results. The model of DUCG can be built in a modular way. This feature makes it easy to build and update complex knowledge bases. The inference process of the DUCG is transparent to doctors, and the diagnostic results are well interpretable, making the diagnostic results more acceptable to clinical doctors. Now, this system has been applied to assist doctors in completing clinical diagnoses in primary hospitals, and it has high diagnostic accuracy.

The rest of this paper is organized as follows. Section 2 introduces the theoretical basis of DUCG, inference methods, and modeling methods of sore throat based on DUCG. Section 3 explains the validation process and results of the diagnostic model of sore throat based on DUCG. Section 4 concludes this paper and outlines future work.

## 2. Materials and Methods

### 2.1. Causal Expression of DUCG

**Figure 1:**
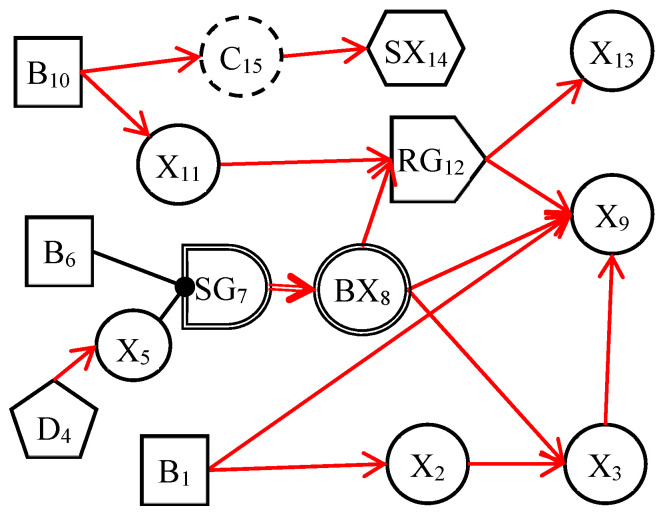
An example of DUCG.

The dynamic uncertain causality graph is a probabilistic graphical model. It can graphically represent the uncertain causalities of events and perform causal reasoning based on the DUCG model [24]. Figure 1 depicts a simple DUCG model. *B*_1_, *B*_6_, and *B*_10_ are root cause events, other variables are consequence events, and they are caused directly or indirectly by these three variables. The red-directed arcs indicate the causal propagation directions. From this DUCG model, we can understand the causal propagation paths among events, such as the causal path (*B*_1_→*X*_2_→*X*_3_→*X*_9_). The variables and their physical meaning in the DUCG are illustrated in Appendix A. When constructing the DUCG model, we can select appropriate types of variables to express knowledge according to the characteristics and functions of events. Different variables play different roles in the causal reasoning process. The DUCG model can be built in a modular way. When building a large and complex DUCG model, we can model some local knowledge as some sub-DUCGs. Then those sub-DUCGs can be automatically merged into a complete DUCG model according to the compilation rules of DUCG [25]. This modular knowledge base construction method reduces the construction difficulty of the large and complex knowledge base and makes the DUCG model well-maintainable. When we need to modify the DUCG model, we only need to modify the local knowledge in the corresponding sub-DUCGs, to achieve the purpose of modifying the whole DUCG model. Some other features of DUCG include: (1) DUCG can deal with loops, so the DUCG model supports the expression of causal loops [26]; (2) DUCG can deal with discrete, continuous, and fuzzy evidence, which increases the robustness of the model [25,27]; (3) the causal reasoning of DUCG depends much on the structure of the model and has low requirements for the precision of model parameters; (4) DUCG can realize the concise expression of knowledge and allow the incomplete expression of knowledge.

**Figure 2:**
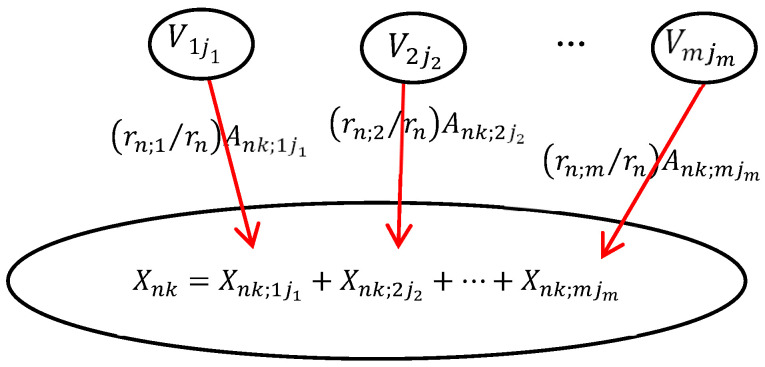
The causal mechanism of the DUCG.

In DUCG, the causal mechanism between a child variable and its parent variables is shown in Figure 2. The child event *X_nk_* may be caused by one or more parent events. In order to calculate the probability that each parent variable causes the occurrence of the child event, the child event performs logic expression expansion operations along the opposite direction of the causal chain. After expression expansion, the child event is expressed by its parent variables. The expansion process can be executed recursively until the parent events are the *B*-type or *BX*-type variables. The *B*-type and *BX*-type variables are the root causes of other variables and the targets of inference calculation. The logic expansion expression is shown in Equation (1).

(1) $$X_{nk}=\sum_{i}\sum_{j_{i}}X_{nk;ij_{i}}=\sum_{i}\sum_{j_{i}}F_{nk;ij_{i}}V_{ij_{i}}=\sum_{i}\sum_{j_{i}}\left(r_{n;i}/r_{n}\right)A_{nk;ij_{i}}V_{ij_{i}}$$

For simplicity, Equation (1) can be briefly written as Equation (2).

(2) $$X_{nk}=\sum_{i}\sum_{j}F_{nk;ij}V_{ij}=\sum_{i}\sum_{j}\left(r_{n;i}/r_{n}\right)A_{nk;ij}V_{ij}$$

In Equation (2), *X_nk_* (*n* is the index of the variable in DUCG, *k* is the current state of *X_n_*, usually, *k* ≠ 0 stands for the abnormal state) denotes the child event. *V_ij_* (*V*∈{*B*, *X*, *BX*, *RG*, *D*, *SG*}) denotes the parent variables of *X_n_*. *F_nk_*_;*ij*_ = (*r_n_*_;*i*_/*r_n_*)*A_nk_*_;*ij*_ is the weighted functional event, the strength of causality that the parent variable *V_ij_* affects the child variable *X_nk_*. *A_nk;ij_* denotes the virtual random functional event representing the causal mechanism that *V_ij_* independently causes *X_nk_*. *r_n_*_;*i*_/*r_n_* ($r_{n}=\sum_{i}r_{n;i}$) is the weight; it is used to normalize the effect of parent variables on child variables.

### 2.2. The Inference Process of the DUCG

The reasoning process of DUCG contains four steps: DUCG simplification, DUCG decomposition, expression expansion, and probability calculation.

Step 1. DUCG simplification. Simplifying the DUCG according to the current evidence *E* (*E* = *E′E″*, *E′* = {X*_ij_*, j ≠ 0} is the collection of abnormal evidence, *E″* = {*X_i_*_0_} is the collection of normal evidence) based on the simplification rules of DUCG. The purposes of simplification are deleting the unrelated variables and causalities under the current evidence and reducing the complexity of inference computation. The simplified DUCG demonstrates the causalities between current evidence and their related hypotheses.

Step 2. Decomposition. The inference of DUCG is based on the rule that abnormal evidence is caused by only one root cause at once. The purpose of decomposition is to decompose the simplified DUCG into a series of sub-DUCGs. The sub-DUCG demonstrates the causalities between a single hypothesis and the current evidence. Meanwhile, we get the hypothesis set *S_H_* = {*H_kj_*} = {*B_kj_*, *BX_kj_*}.

Step 3. Logical expansion of *H_kj_E*. Expand *H_kj_E* according to Equation (2) on each sub-DUCGs. We can get the evidence expansion expressions in the form of sum-of-products composed of only {*B*-, *BX*-, *D*-, *A*-, *r*-}-type events and parameters on each sub-DUCGs, they are used for conditional probability calculation in the next step.

Step 4. Probability calculation. Calculate the evidence probability *ζ_kj_* = Pr{*H_kj_E*} on each sub-DUCG. According to the expansion result of *H_kj_E* in Step 3, *ζ_kj_* can be easily obtained. Then the conditional probability of each hypothesis can be calculated by Equation (3).

(3) $$h_{kj}^{s}=\frac{\zeta_{kj}}{\sum_{k,j}\zeta_{kj}}$$

The results are ranked in descending order as the final inference results.

### 2.3. Sore Throat DUCG Modeling

We cooperated with ENT specialists to construct the sore throat DUCG. The construction of the model not only uses the expert’s clinical knowledge and experience but also uses the results of statistical data [28]. The sore throat DUCG is built in a modular way. We model each disease as one individual sub-DUCG. Then, those sub-DUCGs are merged into one complete DUCG as the knowledge base for sore throat diagnoses. An example of laryngopharyngeal reflux (LPR) illustrates the process of constructing the sub-DUCG.

**Figure 3:**
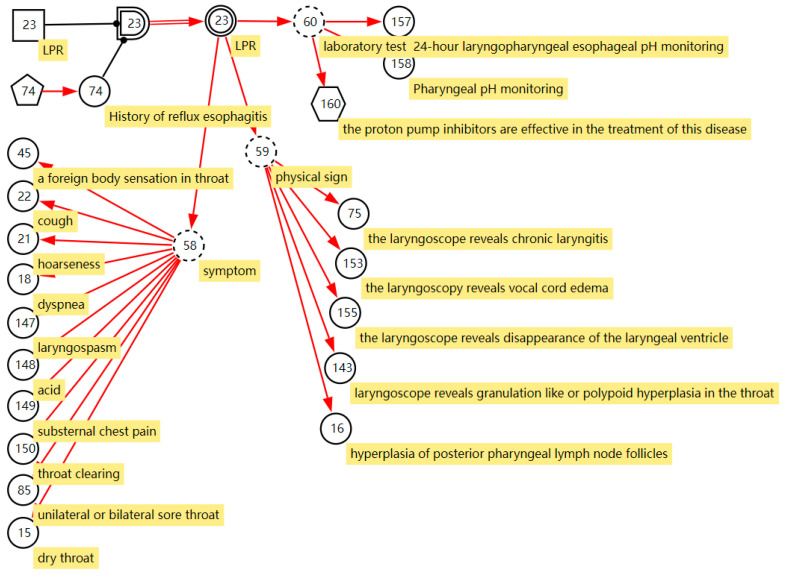
The DUCG of laryngopharyngeal reflux.

LPR is a common disease in otolaryngology. Due to the lack of understanding of the disease in the past, the disease has been misdiagnosed as chronic pharyngitis for a long time. In recent years, as otolaryngologists have gradually deepened their understanding of laryngopharyngeal reflux, they found that the incidence of laryngopharyngeal reflux in the population is very high, accounting for 10% of all patients in otolaryngology outpatient clinics and 50% of patients with hoarseness. The DUCG of LPR is shown in Figure 3. *B*_23_ (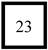) stands for LPR. It has two states; state 0 indicates its negative state and state 1 indicates its positive state with a priori probability of 0.03 (Pr{*B*_23,1_} = 0.03). This probability can be obtained from statistical data of the disease or depending on the experience of the clinical specialists. A history of reflux esophagitis is one risk factor for LPR; people with a history of reflux esophagitis can increase the incidence of LPR 10 times more than people without a reflux esophagitis history. As shown in Figure 3, we use the *X*-type variable numbered *X*_74_ (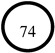) to stand for the history of reflux esophagitis. *SG*_23_ (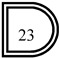) is a special logic gate; its logic specification table records the risk factor combination of *X*_74_ shown in Equation (4) [29]. Equation (4) means that when X_74,1_ is true, state 1 of *SG*_23_ is true (*SG*_23,1_). Otherwise, the state 0 is true (*SG*_23,0_). The different states of *SG*_23_ act with different affections to the incidence of the LPR. The special functional event variable *SA*_23;23_ records the strength of the effect of risk factors on the disease shown in Equation (5).

(4) LGS23=(States of SG23Logic Expression0Remnant1X74,1)

(5) $$SA_{23;23}=\left(\begin{array}{cc} - & -\\ 1 & 10 \end{array}\right)$$

*BX*_23_ (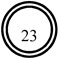) stands for the incidence of the disease when *X*_74,1_ is true, Pr{*BX*_23,1_} = Pr{*SA*_23,1;23,1_*B*_23,1_} = 10 × 0.03 = 0.3. In this way, we express the effect of risk factors on disease incidence in DUCG. Manifestations caused by LPR are drowning as the children of the *BX*-type variable; most of them are represented by the X-type variable standing for the nonspecific clinical manifestations, except the manifestation “proton pump inhibitors are effective in the treatment of this disease”. The manifestation *SX*_160_ (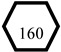, proton pump inhibitors are effective in the treatment of this disease) is the clinical gold standard for LPR diagnosis; it is represented by the *SX*-type variable. When the manifestation appears, the disease can be directly diagnosed based on this evidence. Appendix B shows the parameters of causal strength between variables of the sub-DUCG of LPR shown in Figure 3. This sub-DUCG model describes the relationship between LPR and its clinical diagnostic information, including the involved symptoms, signs, laboratory tests, diagnostic gold standard, risk factors, and other information. This information is understandable to doctors.

**Figure 4:**
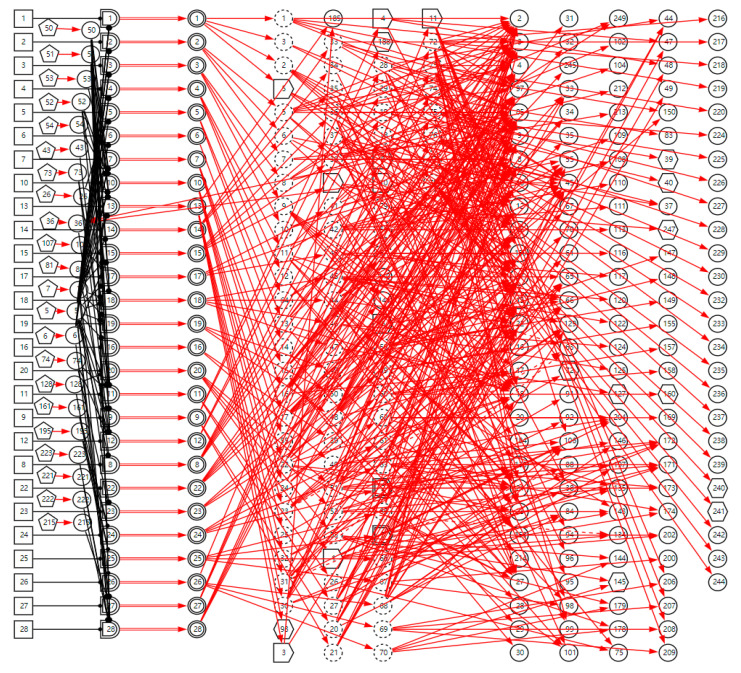
The complete DUCG of sore throat is used as the diagnostic knowledge base for sore throat.

The complete DUCG with a sore throat as the chief complaint is shown in Figure 4. Currently, it contains 27 diseases, including acute and chronic inflammation, trauma, cancer, and other diseases related to sore throat; the diseases are shown in Table 1. A total of 354 variables are used to build the DUCG, 27 groups of {*B*, *SG*, *BX*}-type variable combinations are used to represent diseases and the impact of risk factors on diseases. A total of 153 *X*-type variables are divided into two classes, 22 variables are used to stand for the risk factors, 131 variables are used to represent nonspecific clinical manifestations, and 11 *SX*-type variables stand for the specific clinical manifestations. A total of 76 *C*-type variables are used to classify the diseases’ manifestations in each sub-DUCG. A total of 651 *F*-type variables are used to represent the causalities between variables. As we can see, the complete DUCG is complex, and it is difficult for medical specialists to build this knowledge base directly on one graph. The modular knowledge base construction method of DUCG makes the construction of large and complex knowledge bases feasible and simple.

## 3. Results

The computer-aided diagnostic model based on DUCG has good interactivity and interpretability. Doctors can make clinical inquiries based on diagnoses and carry out the following diagnosis until the disease is confirmed. A case is employed to explain the diagnostic process of DUCG.

A young (*X*_7,4_) male (*X*_52,1_) patient with bilateral sore throat (*X*_85,1_) as the chief complaint, together with the symptoms of hoarseness (*X*_21,1_), foreign body sensation in throat (*X*_45,1_), throat itching (*X*_44,1_), throat clearing (*X*_150,1_), subacute stage (*X*_5,1_), other symptoms that need to be consulted are negative, i.e., dry throat (*X*_51,0_), cough (*X*_22,0_), expectoration (*X*_23,0_), dyspnea (*X*_18_,_0_). When we input the evidence *E* = *E′E″* into the model (*E′* = *X*_7,4_*X*_52,1_*X*_85,1_*X*_21,1_*X*_15,1_*X*_45,1_*X*_44,1_*X*_150,1_*X*_5,1_ is the positive symptoms of the patient, *E″* is the negative symptoms of the patient). The top 5 inference results are shown in Table 2, and the probabilities of other diseases are less than 1%.

**Figure 5:**
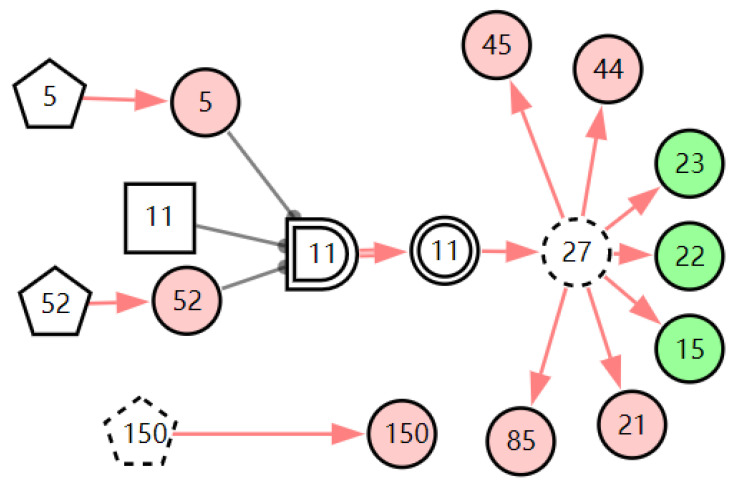
The graphic interpretation of chronic laryngitis when *E′* = *X*_7,4_*X*_52,1_*X*_85,1_*X*_21,1_*X*_15,1_*X*_45,1_*X*_44,1_*X*_150,1_*X*_5,1_.

**Figure 6:**
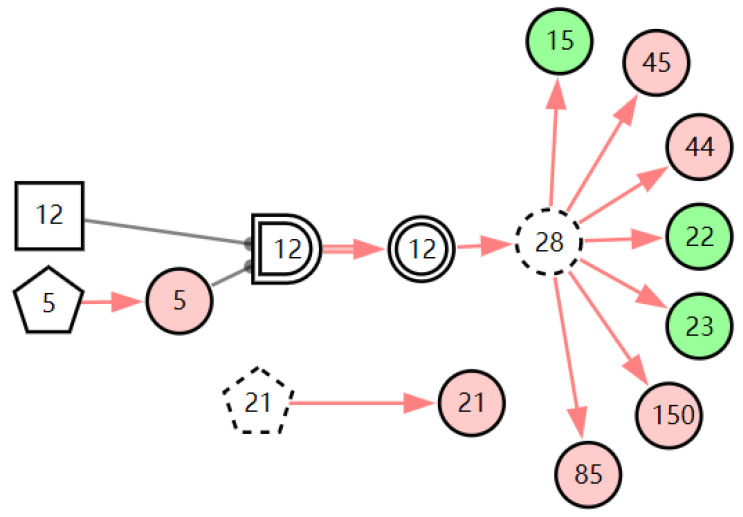
The graphic interpretation of chronic pharyngitis when *E′* = *X*_7,4_*X*_52,1_*X*_85,1_*X*_21,1_*X*_15,1_*X*_45,1_*X*_44,1_*X*_150,1_*X*_5,1_.

**Figure 7:**
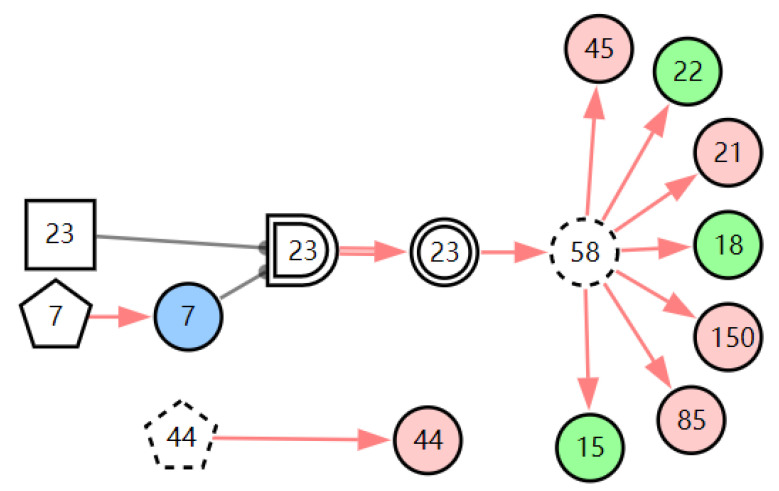
The graphic interpretation of LPR when *E′* = *X*_7,4_*X*_52,1_*X*_85,1_*X*_21,1_*X*_15,1_*X*_45,1_*X*_44,1_*X*_150,1_*X*_5,1_.

According to the patient’s current symptoms, the inference results of the DUCG show that the patient is most likely to have chronic laryngitis. Chronic pharyngitis comes second, and LPR comes third. The patient is less likely to suffer from other diseases. Figure 5, Figure 6 and Figure 7 are graphic interpretations of the three diseases. From Figure 5, we can see that chronic laryngitis can explain most abnormal symptoms, except for the evidence of throat clearing (*X*_150,1_). Throat clearing is not the manifestation of chronic pharyngitis. Therefore, it is regarded as isolated evidence in the Figure. Standing for it cannot be explained by the current disease. It decreases the conditional probability of the disease during the reasoning calculation. *X*_15,0_, *X*_23,0_, and *X*_22,0_ are normal evidence; they function as negative evidence to reduce the conditional probability of the disease. *X*_5,1_ and *X*_52,1_ are two risk factors for chronic laryngitis. They increase the incidence of the disease. Similarly, chronic pharyngitis and laryngopharyngeal reflux also have isolated and normal evidence. In the DUCG model, the prior probabilities of the three diseases are 0.04, 0.09, and 0.03. Therefore, the diagnostic result is reasonable based on the current evidence, and the diagnostic results provide a reference for follow-up consultation and physical examination.

**Figure 8:**
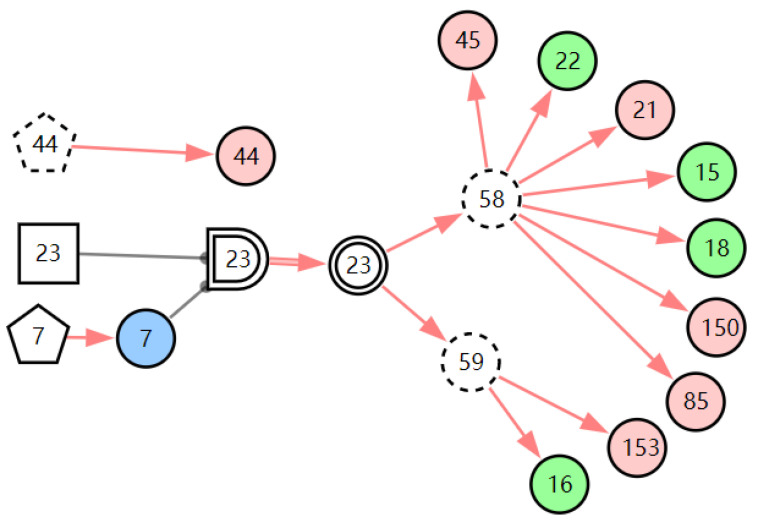
The graphic interpretation of LPR when *E′* = *X*_7,4_*X*_52,1_*X*_85,1_*X*_21,1_*X*_15,1_*X*_45,1_*X*_44,1_*X*_150,1_*X*_5,1_*X*_153,1_.

**Figure 9:**
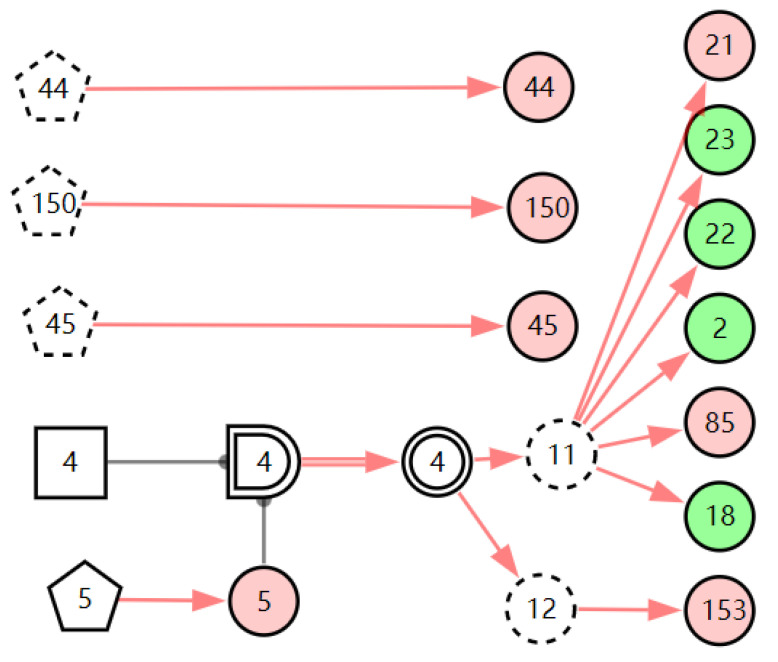
The graphic interpretation of acute laryngitis when *E′* = *X*_7,4_*X*_52,1_*X*_85,1_*X*_21,1_*X*_15,1_*X*_45,1_*X*_44,1_*X*_150,1_*X*_5,1_*X*_153,1_.

**Figure 10:**
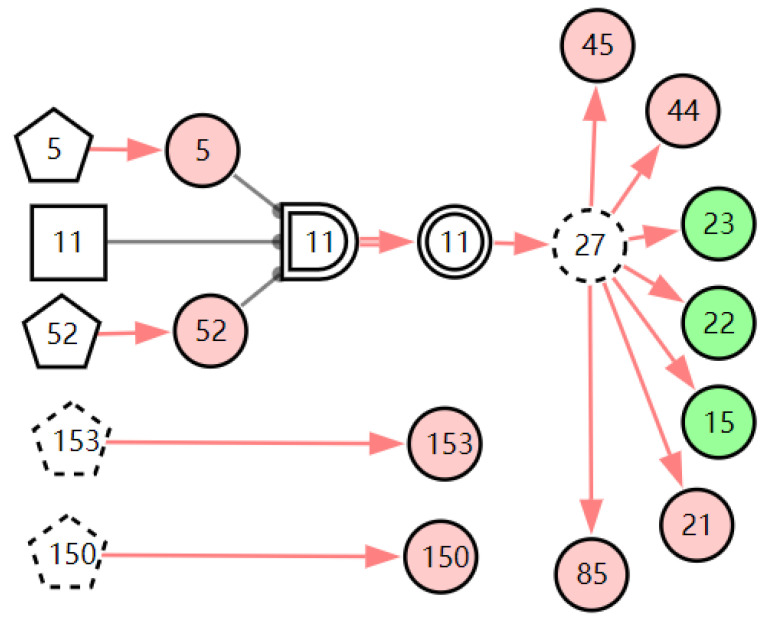
The graphic interpretation of chronic laryngitis when *E′* = *X*_7,4_*X*_52,1_*X*_85,1_*X*_21,1_*X*_15,1_*X*_45,1_*X*_44,1_*X*_150,1_*X*_5,1_*X*_153,1_.

According to the first diagnostic result, the physical signs related to these three diseases were checked first. Physical examination found that the patient has one positive physical sign; laryngoscopy reveals vocal cord edema. The evidence *E* = *E′E″*(*E′* = *X*_7,4_*X*_52,1_*X*_85,1_*X*_21,1_*X*_15,1_*X*_45,1_*X*_44,1_*X*_150,1_*X*_5,1_*X*_153,1_) is inputted into the model for another diagnosis, and the diagnostic results are shown in Table 3. The probability of LPR is 82.74%. The probabilities of acute laryngitis and chronic laryngitis are only 11.11% and 6.11%. They are far less than the probability of LPR. From the graphic interpretation in Figure 8, Figure 9 and Figure 10, we can see that LPR can explain the patient’s abnormal physical signs. Although acute laryngitis can explain abnormal physical signs, it has 3 unexplainable abnormal symptoms. Similarly, chronic laryngitis can not explain abnormal physical signs. We can initially confirm that the patient has LPR, depending on the diagnostic result. In the following, some laboratory tests or imaging tests related to LPR are done to validate the result.

**Figure 11:**
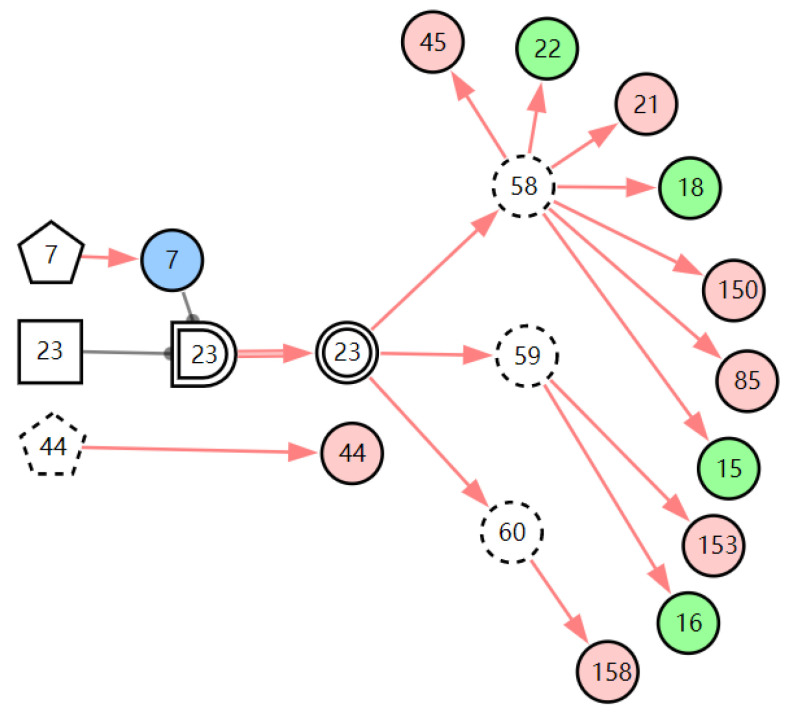
The graphic interpretation of LPR when *E′* = *X*_7,4_*X*_52,1_*X*_85,1_*X*_21,1_*X*_15,1_*X*_45,1_*X*_44,1_*X*_150,1_*X*_5,1_*X*_153,1_*X*_158,1_.

In the case record, the patient’s routine blood test report showed that the patient’s neutrophil percent (NEUT%) was normal (*X*_9,0_) and the white blood cell count (WBC) was normal (*X*_8,0_). The result of pharyngeal pH monitoring was positive (*X*_158,1_). When we inputted this new evidence *E* = *X*_158,1_*X*_9,0_*X*_8,0_ to the model, the diagnostic results showed that the probability of LPR is 99.98%. From the graphic interpretation in Figure 11, we can see that LPR can explain all the abnormal evidence except throat itching. This means most of the abnormal evidence can be traced back to LPR, so the diagnostic result is believable. Throat itching is not the clinical manifestation of LPR; it is regarded as interference with the diagnosis of LPR. The existence of interference evidence does not affect the diagnostic results of the model, which shows that the model has good robustness.

This case study demonstrates the whole diagnostic process of the DUCG. The disease is finally diagnosed through a gradual process of continuous inference and clinical inquiries. Based on the diagnostic result in each step, the scope of the disease is determined. Further consultation information for each disease can be calculated based on the DUCG. The graphical explanation can explain every step of the calculation so that the doctor can understand the whole reasoning process of the system, and it is convenient for the doctor to make a judgment on rejecting or accepting the reasoning results of the system.

The validation of the model contains two stages. First, the creator of the knowledge base self-tests the model. The purpose of the self-test is to initially verify the correctness of knowledge representation in the model and adjust the knowledge structure of the model according to the test results. The test cases are selected from published case reports, outpatient cases, or created by the medical specialists by their experience. For the diseases in the DUCG model, each disease was tested with 3 cases, and a total of 81 cases were used to test the model. The accuracy of the test was 100%. The self-test results manifest that the medical knowledge expression of the model is reasonable and correct. If the test finds that the knowledge expression is wrong, the medical specialist should modify the model. After modifying the model, the original case and some new cases are used to test the model again to avoid the overfitting problem.

The second stage of testing is third-party testing. The third-party hospital is Suining Central Hospital, a Grade 3 and Class A hospital located in Suining City, Sichuan Province. During the test, the doctor reads the clinical information in the case, inputs it into the system for calculation, and compares whether the calculation results of the system are consistent with the case record results. The test cases are randomly selected from the health information system (HIS) of the hospital from the past five years. The test cases were obtained using an equal sampling method. Each disease is tested with 10 cases. If there are fewer than 10 cases of the disease, all eligible cases are used to test for the disease. The test results are shown in Table 4.

In the HIS of Suining Central Hospital, in the past five years, a total of 2592 cases can be used to test the diagnosis model of pharyngeal pain, among which there are more common inflammatory diseases and fewer tumor-related diseases. A total of 196 cases with sore throat as their chief complaint was used to test the model, accounting for 7.5% of the total cases. For each case, the doctor reads the patient’s clinical information recorded in the case and inputs it into the system. The system makes clinical diagnoses according to the input information and outputs the probability of each disease the patient may have in the form of probabilities. The top 1 disease is regarded as the system’s diagnostic result. Doctors compare the diagnosed diseases recorded in the cases with the system results. If the results are consistent, the system’s diagnostic result is true (true case); otherwise, the diagnosis is considered false (false case). The accuracy of the diagnostic system is evaluated by Equation (6).

(6) $$Accuracy=\frac{true\;\mathrm{cases}}{test\;\mathrm{cases}}\times 100\%$$

Out of 196 cases, 194 cases were correctly diagnosed, and the diagnostic accuracy was 98.9%. Two cases were misdiagnosed. One infectious mononucleosis case was misdiagnosed as acute tonsillitis. Another case is peritonsillitis, which was misdiagnosed as chronic pharyngitis. Three diseases (pharyngeal burn, laryngeal syphilis, and pharyngeal syphilis) are not validated because there have been no cases in the HIS of the hospital in the past five years.

After the third-party test, the model was used for clinical assistant diagnoses in all primary hospitals in Jiaozhou City, Shandong Province, China. In clinical diagnoses, the doctor inputs the patient’s self-reported symptoms and physical signs into the system for preliminary calculation. For some common diseases, if the doctor highly agrees with the diagnostic result, then the diagnosis is completed, and the doctor evaluates the diagnostic results of the system. For some uncommon diseases, such as cancer, the doctor should input the patient’s symptoms and physical signs for initial diagnoses and advise the patient to perform corresponding imaging or laboratory tests. Then, all the evidence is input into the system for diagnoses. This result is used as the final diagnostic result. The actual application of the model is shown in Table 5.

Table 5 is the application data of the system from 8 April 2020 to 16 April 2022. In the past two years, doctors used the system to diagnose 7236 patients with sore throat, involving a total of 16 conditions. In the process of using the system, we collected doctors’ feedback on the recognition of diagnostic results. Doctors’ recognition of the diagnostic system exceeded 99.9%. Among the 7236 diagnostic results, doctors had doubts about the diagnostic results only once. This shows that it is feasible to use the system for clinical assistant diagnoses in primary hospitals.

## 4. Conclusions

Doctors in primary hospitals have the problem of a lack of diagnostic knowledge and insufficient experience, which is the main reason for missed diagnoses and misdiagnoses. In this study, we develop a computer-aided diagnostic system for differential diagnoses of sore throats based on DUCG. The diagnostic model integrates medical specialists’ knowledge, experience, and epidemiological data and presents the diagnostic knowledge of diseases in a way that doctors can intuitively understand. The purpose of designing the diagnostic system is to help doctors make differential diagnoses of sore throat-related diseases and reduce misdiagnoses and missed diagnoses caused by lacking knowledge and experience. Meanwhile, we hope doctors can improve their diagnostic experience and knowledge using the system.

The diagnostic accuracy of the model depends on the accuracy and completeness of expert clinical diagnosis and knowledge expression. Therefore, this study’s main challenge is building a large and complex diagnostic model and ensuring the accuracy of knowledge expressed in the model. Building a knowledge base with experienced clinical experts and verifying the knowledge base many times is one method to ensure the accurate expression of knowledge. With the help of DUCG’s modular model construction method and causal knowledge expression method, each disease is constructed as an independent sub-DUCG model, which can be understood and maintained easily. The reasoning mode of DUCG is chain reasoning; that is, based on the current evidence, the evidence along the causal propagation chain is expanded until it reaches the root cause variable, then the conditional probability of each hypothesis under the current evidence is calculated, and the results are explained graphically. This reasoning method is in line with the diagnostic idea of evidence-based medicine in clinical science and is easily accepted by doctors.

The model was built by medical specialists in otolaryngology at Capital Medical University Xuanwu Hospital and can differentially diagnose 27 common and uncommon sore throat-related diseases. The model used 81 cases from Xuanwu Hospital for self-test, and the test accuracy was 100%. Then, the model was tested by a third party, and the test accuracy was 99.8%. Currently, the model has been applied in primary hospitals in Jiaozhou City, Shandong Province. Doctors agree with the diagnosis results by more than 99.9%. This shows that it is feasible to use DUCG for sore throat-related diseases and has high diagnostic accuracy. It can be applied to primary hospitals to assist doctors in clinical diagnosis. Meanwhile, the study indicates that it is feasible to construct a diagnostic model based on expert knowledge, experience, and statistical data.

The purpose of this study is to differentially diagnose the diseases with a sore throat as the chief complaint, that is, the patient with a sore throat as his main symptom. If the patient has no sore throat or it is not his main symptom, this diagnostic model is not applicable. In order to realize clinical assistant diagnoses in general practice, we construct many diagnostic models according to different chief complaints. For example, the model of abdominal pain takes abdominal pain as the chief complaint and can diagnose 93 kinds of diseases related to abdominal pain. At present, we have constructed 46 diagnostic models with different chief complaints, and these models have been applied in clinical practice. In clinical diagnoses, the doctor chooses the corresponding diagnostic model according to the patient’s chief complaint, then inputs the patient’s clinical information for diagnoses. We constantly update and improve the diagnostic system according to the doctors’ feedback. There are two kinds of improvement. The first is the improvement of the chief complaint. If the doctors propose to add a new chief complaint model, we will build a new DUCG model based on the chief complaint, and all diseases in the model will take the chief complaint as the main symptom. The second part is the updating and improvement of the model. If doctors find a disease missing in the model, we will add the disease to the model so that the model can diagnose this disease. Based on the DUCG modular modeling approach, this model is easily updated. We plan to use this approach to improve the disease diagnosis capabilities of the system continuously. In the following work, we will continue to expand the model so that the model can diagnose more diseases. In addition, we consider adding treatment guidelines to the model so that the model can recommend treatment for doctors after getting the diagnostic conclusion.

## Figures and Tables

**Table 1:** The sore throat-related diseases in the complete DUCG.

Classification	Disease	ID
Inflammation	Acute tonsillitis	*B*_1_
	Acute pharyngitis	*B*_2_
	Acute epiglottitis	*B*_3_
	Acute laryngitis	*B*_4_
	Chronic laryngitis	*B*_11_
	Chronic pharyngitis	*B*_12_
	Chronic tonsillitis	*B*_9_
	Peritonsillitis	*B*_19_
	Peritonsillar abscess	*B*_7_
Trauma	Pharyngeal burn	*B*_5_
	Closed laryngeal trauma	*B*_17_
Foreign body	Pharyngeal foreign body	*B*_8_
Tumor	Cancer of the larynx	*B*_10_
	Tonsil carcinoma	*B*_13_
	Carcinoma of hypopharynx	*B*_18_
	Tonsil lymphoma	*B*_22_
Tuberculosis	Laryngeal tuberculosis	*B*_20_
	Pharyngeal tuberculosis	*B*_26_
Syphilis	Laryngeal syphilis	*B*_24_
	Pharyngeal syphilis	*B*_25_
Uncommon disease	Glossopharyngeal neuralgia	*B*_6_
	Styloid process syndrome	*B*_14_
	Infectious mononucleosis	*B*_15_
Cardiovascular disease	Coronary heart disease	*B*_28_
Reflux disease	Laryngopharyngeal reflux	*B*_23_
Bacterial or viral infection	Upper respiratory tract infection	*B*_27_
	Throat ulcers	*B*_16_

**Table 2:** The diagnostic result of DUCG basing on the patient’s current symptoms.

Disease	ID	Probability
Chronic laryngitis	*B*_11,1_	39.52%
Chronic pharyngitis	*B*_12,1_	26.56%
Laryngopharyngeal reflux	*B*_23,1_	25.50%
Chronic tonsillitis	*B*_9,1_	5.89%
Acute laryngitis	*B*_4,1_	1.92%

**Table 3:** The diagnostic result of DUCG basing on the patient’s current symptoms and physical signs.

Disease	ID	Probability
Laryngopharyngeal reflux	*B*_23,1_	82.74%
Acute laryngitis	*B*_4,1_	11.11%
Chronic laryngitis	*B*_11,1_	6.11%
Chronic pharyngitis	*B*_12,1_	0.017%
Acute epiglottitis	*B*_3,1_	0.0039%

**Table 4:** The third-party test of the model in Suining Central Hospital.

Disease Name	Total Cases	Test cases	True Cases	Accuracy
Acute tonsillitis	388	10	10	100%
Acute pharyngitis	129	10	10	100%
Acute epiglottitis	233	10	10	100%
Acute laryngitis	204	10	10	100%
Pharyngeal burn	0	0	0	0%
Glossopharyngeal neuralgia	6	4	4	100%
Peritonsillar abscess	26	10	10	100%
Pharyngeal foreign body	11	10	10	100%
Chronic tonsillitis	831	10	10	100%
Cancer of the larynx	55	10	10	100%
Chronic laryngitis	14	9	9	100%
Chronic pharyngitis	255	10	10	100%
Throat ulcers	45	9	9	100%
Tonsil carcinoma	4	4	4	100%
Styloid process syndrome	2	2	2	100%
Infectious mononucleosis	53	10	9	90%
Closed laryngeal trauma	8	8	8	100%
Carcinoma of hypopharynx	15	10	10	100%
Peritonsillitis	120	10	9	90%
Laryngeal tuberculosis	14	10	10	100%
Tonsil lymphoma	1	1	1	100%
Laryngopharyngeal reflux	6	5	5	100%
Laryngeal syphilis	0	0	0	0%
Pharyngeal syphilis	0	0	0	0%
Pharyngeal tuberculosis	2	2	2	100%
Upper respiratory tract infection	157	10	10	100%
Coronary heart disease	13	9	9	100%
Total	2592	196	194	98.96%

**Table 5:** Application of sore throat diagnostic model in primary hospitals in Jiaozhou City.

Disease	Diagnosed Cases	Agreed Diagnoses
Pharyngeal foreign body	48	48
Throat ulcers	55	55
Carcinoma of hypopharynx	3	3
Glossopharyngeal neuralgia	18	18
Upper respiratory tract infection	2625	2625
Chronic pharyngitis	564	564
Chronic laryngitis	152	152
Chronic tonsillitis	65	65
Acute pharyngitis	1188	1188
Acute epiglottitis	809	809
Chronic laryngitis	907	906
Acute tonsillitis	425	425
Coronary heart disease	325	325
Laryngopharyngeal reflux	29	29
Peritonsillitis	17	17
Peritonsillar abscess	6	6
Total	7236	7235

## Data Availability

The data presented in this study are available on request from the corresponding author. The data are not publicly available due to the medical record data involves patient privacy.

## Appendix A

The variables and their physical meaning in the DUCG.

The root cause variable (B-type variable, written as *B_n_*, represented as ) is used to represent the root cause that causes other variables to occur.

The consequence or process variable (X-type variable, written as *X_n_*, represented as ), which is used to represent the result caused by the root cause variable, can also be used as the cause of other variables.

The special manifestation of a root cause variable (SX-type variable, written as *SX_n_*, represented as ), only connects with one root cause variable. If it is true, then the corresponding root cause must be true.

The unknown cause or default cause variables (D-type variable, written as *D_n_*, represented as ), when the cause of a variable’s occurrence is unknown, then a *D*-type variable is used to represent the root cause variable that causes it to occur.

The special logic gate (SG-type variable, written as *SG_n_*, represented as ) is only used to express logical relationships between the *B*-type variable and *X*-type variables.

The integrated cause variable (BX-type variable, written as *BX_n_*, represented as ) integrates multiple root cause events as one cause event. It expresses the joint affection of multiple causes.

The classification variable (C-type variable, written as *C_i_*, represented as ) can organize the variable by group, making the DUCG more readable and understandable.

The reverse logic gate (RG-type variable, written as *RG_n_*, represented as ) is used to represent the logic combinations of its child variables.

The red-directed arc (F-type variable, written as *F_n_*_;*i*_, represented as →) is the weighted functional event variable; it is used to represent and quantify the causalities between parent variables and child variables. The red dashed directed arc is the condition weighted functional event variable; its condition is described as *Z_i;j_*. When *Z_i;j_* is true, the causal relationship between its parent and child variable holds. Otherwise, the causality does not exist.

The double line directed arc (SA-type variable, written as *SA_n_*_;*I*_, represented as ) is the special functional event variable. It is only used between the *SG*-type variable and *BX*-type variable, quantifying the joint affection of the variable combinations in the logical gate specification of the SG-type variable.

## Appendix B

The parameters of causal strength between variables in the DUCG of the LPR. The parameter *b_i_* records the prior probability of abnormal states of diseases. The parameter *a_i_*_;*j*_ records the causal strengths that the variable *V_j_* may cause *V_i_* to occur. The parameter $\varepsilon_{i,j}$ is the importance of variables, which is used to calculate the influence of isolated evidence on reasoning. The accuracy of these parameters is not strictly required. Doctors only need to give the relative magnitude of the action intensity between variables, while reasoning diagnosis has a high dependence on the model structure and low dependence on parameters.

$$\begin{array}{l} b_{23}=\left(\begin{array}{cc} - & 0.03 \end{array}\right),\;a_{60;23}=\left(\begin{array}{cc} 1 & 0\\ 0 & 1 \end{array}\right),\;a_{157;60}=\left(\begin{array}{cc} - & -\\ - & 0.95 \end{array}\right),\;a_{158;60}=\left(\begin{array}{cc} - & -\\ - & 0.9 \end{array}\right),\;\\ a_{160;60}=\left(\begin{array}{cc} - & -\\ - & 0.9 \end{array}\right),\;a_{59;23}=\left(\begin{array}{cc} 1 & 0\\ 0 & 1 \end{array}\right),\;a_{75;59}=\left(\begin{array}{cc} - & -\\ - & 0.85 \end{array}\right),\;a_{153;59}=\left(\begin{array}{cc} - & -\\ - & 0.4 \end{array}\right),\;\\ a_{155;59}=\left(\begin{array}{cc} - & -\\ - & 0.5 \end{array}\right),\;a_{143;59}=\left(\begin{array}{cc} - & -\\ - & 0.5 \end{array}\right),\;a_{16;59}=\left(\begin{array}{cc} - & -\\ - & 0.3 \end{array}\right),\;a_{58;23}=\left(\begin{array}{cc} 1 & 0\\ 0 & 1 \end{array}\right),\;\\ a_{45;58}=\left(\begin{array}{cc} - & -\\ - & 0.8 \end{array}\right),\;a_{22;58}=\left(\begin{array}{cc} - & -\\ - & 0.7 \end{array}\right),\;a_{21;58}=\left(\begin{array}{cc} - & -\\ - & 0.8 \end{array}\right),\;a_{18;58}=\left(\begin{array}{cc} - & -\\ - & 0.1 \end{array}\right),\;\\ a_{147;58}=\left(\begin{array}{cc} - & -\\ - & 0.4 \end{array}\right),\;a_{148;58}=\left(\begin{array}{cc} - & -\\ - & 0.6 \end{array}\right),\;a_{149;58}=\left(\begin{array}{cc} - & -\\ - & 0.4 \end{array}\right),\;a_{150;58}=\left(\begin{array}{cc} - & -\\ - & 0.9 \end{array}\right),\;\\ a_{15;58}=\left(\begin{array}{cc} - & -\\ - & 0.3 \end{array}\right),\;a_{85;58}=\left(\begin{array}{cc} - & -\\ - & 0.4\\ - & 0.4 \end{array}\right),\;a_{74;74D}=\left(\begin{array}{cc} - & 0.05 \end{array}\right),\;r_{n;i}=1,\;\varepsilon_{15,1}=15,\;\\ \varepsilon_{16,1}=20,\;\varepsilon_{18,1}=40,\;\varepsilon_{21,1}=30,\;\varepsilon_{22,1}=25,\;\varepsilon_{45,1}=25,\;\varepsilon_{75,1}=99,\;\varepsilon_{85,1}=\varepsilon_{85,2}=10,\;\\ \varepsilon_{143,1}=75,\;\varepsilon_{147,1}=30,\;\varepsilon_{148,1}=25,\;\varepsilon_{149,1}=30,\;\varepsilon_{150,1}=15,\;\varepsilon_{153,1}=75,\;\varepsilon_{155,1}=85,\;\\ \varepsilon_{157,1}=80,\;\varepsilon_{158,1}=75,\;\varepsilon_{160,1}=60. \end{array}$$
